# *Lycium barbarum* glycopeptide protects neurons from amyloid-beta toxicity

**DOI:** 10.4103/NRR.NRR-D-25-00501

**Published:** 2025-10-30

**Authors:** Zihang Chen, Xiao Wang, Simin Wang, Zelin Wu, Huajun Wang, Kin Chiu, Xiaofei Zheng, Kwok-Fai So

**Affiliations:** 1 Department of Sports Medicine, The First Affiliated Hospital, Guangdong Provincial Key Laboratory of Speed Capability, The Guangzhou Key Laboratory of Precision Orthopedics and Regenerative Medicine, Jinan University, Guangzhou, Guangdong Province, China; 2 Department of Psychology, State Key Laboratory of Brain and Cognitive Sciences, The University of Hong Kong, Hong Kong Special Administrative Region, China; 3 Guangdong-Hongkong-Macau Institute of Central Nervous System (CNS) Regeneration, Ministry of Education Central Nervous System (CNS) Regeneration Collaborative Joint Laboratory, Jinan University, Guangzhou, Guangdong Province, China; 4 Department of Anesthesiology, Zhongshan Hospital of Traditional Chinese Medicine Affiliated to Guangzhou University of Traditional Chinese Medicine, Zhongshan, Guangdong Province, China

**Keywords:** Alzheimer’s disease, amyloid-beta, brain-derived neurotrophic factor, *Lycium barbarum* glycopeptide, N2A cells, neuroinflammation, neuroprotection, oxidative stress, PI3K/Akt/p70S6K signaling, reactive oxygen species

## Abstract

Oligomeric amyloid-beta deposition within neurons and its associated neurotoxicity are among the most direct indicators of the onset of Alzheimer’s disease. Investigations into strategies aimed at reducing amyloid-beta accumulation or promoting its clearance in neurons are currently being carried out. *Lycium barbarum* glycopeptide is rich in biologically active compounds and possesses antioxidant, anti-inflammatory, and neuroprotective properties. This study verified that the oligomer amyloid-beta1–42 induced toxic effects in mouse N2a neuroblastoma cells, resulting in elevated levels of reactive oxygen species and increased expression of the inflammatory enzyme inducible nitric oxide synthase. *Lycium barbarum* glycopeptide counteracted these effects by alleviating cell damage, enhancing cell viability, reducing the levels of reactive oxygen species and inducible nitric oxide synthase expression, and up-regulating the mRNA levels of antioxidant enzymes, including superoxide dismutase 1, superoxide dismutase 2, and glutathione peroxidase 4. *Lycium barbarum* glycopeptide also activated the phosphoinositide 3-kinase/Akt/p70S6K signaling pathway and promoted the expression of brain-derived neurotrophic factor, thus exerting neuroprotective effects. These findings indicate that *Lycium barbarum* glycopeptide may be a promising candidate for the treatment of Alzheimer’s disease.

## Graphical abstract

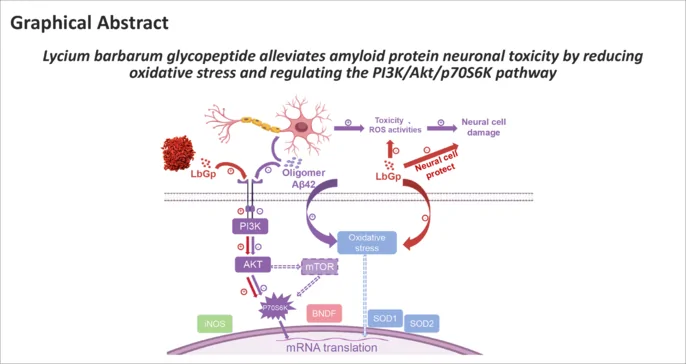

## Introduction

Patients with Alzheimer’s disease (AD) exhibit cognitive dysfunction and memory loss (Marvi et al., 2025). The formation of extracellular senile plaques and intracellular neurofibrillary tangles are pathological hallmarks of the disease. There are several hypotheses regarding the pathogenesis of AD, including the amyloid cascade (Kepp et al., 2023), tau protein (Zheng et al., 2024), inflammation (Hanslik and Ulland 2020), metal ion (Chen et al. 2023), and oxidative stress hypotheses (Bai et al., 2022). Numerous studies have shown significant accumulation of reactive oxygen species (ROS) and increased oxidative stress in patients with AD, including the activation and overexpression of related oxidases (Ganguly et al., 2021; Ionescu-Tucker and Cotman, 2021; Choi et al., 2024). Amyloid-beta (Aβ) protein, as one of the primary pathogenic mechanisms of AD, has been proven to promote ROS increase and induce oxidative stress, causing neuronal cell damage (Bai et al., 2022). In the amyloidogenic pathway, amyloid precursor protein is cleaved by β-secretase and γ-secretase, producing Aβ_1–40_ and Aβ_1–42_ peptides, which are then transported to the extracellular region via receptors, where they aggregate to form oligomeric Aβ (OAβ). OAβ in turn impairs the activity of N-methyl-D-aspartic acid receptor subtypes, leading to the production of extracellular ROS and excessive calcium influx into neurons, ultimately causing mitochondrial dysfunction (Sutherland et al., 2013). The aggregation of OAβ also produces Aβ fibers and extracellular senile plaques, leading to oxidative stress and promoting cell apoptosis (Cheignon et al., 2018). Strategies such as the direct removal of free radicals through oxidation (Browne et al., 2019), activation of endogenous antioxidant pathways (Dinkova-Kostova et al., 2018), and inhibition of metal ion-mediated oxidative damage (Takeda and Tamano, 2021) have demonstrated promising outcomes in animal models, and ameliorating oxidative stress and improving neuroprotection thus represent promising approaches for enhancing the efficacy of AD treatments.

The mouse neural crest-derived Neuro-2a (N2a) neuroblastoma cell line has been widely employed as a model to investigate neuronal differentiation, neurite outgrowth, synaptogenesis, signaling pathways, and neuroprotection (Gao et al., 2020). N2a cells have the advantage of rapid responsiveness to environmental stimuli via the expression of signaling molecules that drive neuronal differentiation and neurite extension (Tremblay et al., 2010). In relation to AD research, N2a cells provide a reproducible and easily manipulated system to assess Aβ-induced toxicity, including oxidative stress, apoptosis, and mitochondrial dysfunction (Wang et al., 2022; Wang and Jia, 2023). however, similar to other cell experiments, this *in vitro* research has several limitations. No single model can fully replicate the pathology of human AD, because humans demonstrate a more intricate disease progression, encompassing tau pathology and contributions of the vascular system to neurodegeneration. The phosphoinositide 3-kinase (PI3K)/Akt/mechanistic target of rapamycin (mTOR)/ribosomal protein S6 kinase beta-1 (p70S6K) axis serves as a fundamental regulatory mechanism governing axonal and dendritic morphogenesis, facilitating cytoskeletal protein biosynthesis, including activity-regulated cytoskeleton-associated protein (Kwon et al., 2003). Accumulating evidence (Lafay-Chebassier et al., 2005) has demonstrated persistent down-regulation of mTOR/p70S6K signaling activity across various experimental models, including neuroblastoma cells exposed to Aβ, cortical neurons derived from amyloid precursor protein/presenilin 1 transgenic mice, and lymphocytes from patients with AD. Further mechanistic investigations have revealed that even low Aβ concentrations are sufficient to induce functional impairment of the PI3K/Akt/mTOR signaling pathway, highlighting its potential dysregulation in AD-related neurodegeneration (Pei and Hugon, 2008).

*Lycium barbarum* has been used in China for over 2000 years as a traditional medicinal herb and dietary supplement., and *Lycium barbarum* polysaccharide (LBP) exhibits various activities, including antioxidative, anti-aging, and anti-inflammatory effects, reproductive protection, vision support, and immune modulation (Dai et al., 2023; Jiang et al., 2024; Xiong et al., 2025). *Lycium barbarum* glycopeptide (LbGp) is a further extract of LBP, consisting of five sugar conjugates connected by glycopeptides (LbGP1–LbGP5), collectively referred to as LbGp (Tian et al., 1995). LbGp has multiple biological activities, showing significant neuroprotective effects. For instance, LbGp effectively counteracted oxidative stress and lipid peroxidation in the cerebral cortex of an anxiety mouse model, reducing its toxic effects on neuronal cells (Dai et al., 2023). LbGp has also been shown to attenuate the inflammatory response in microglia, likely via inhibition of the mitogen-activated protein kinase (MAPK)/nuclear factor-κB (NF-κB) signaling pathway and pyroptosis, which in turn facilitated neural repair and the restoration of motor functions in rodent models of spinal cord injury (Jiang et al., 2023). It can also ameliorate depression induced by aversive stimuli by inhibiting microglial cell activation (Fu et al., 2021). Recent research also found that LbGp not only possessed anti-aging properties by regulating glucagon-like peptide-1 and the oxidative stress molecule SKN-1, but also improved neurodegenerative diseases such as Parkinson’s disease in a DAF-16-, SKN-1-, and heat shock factor 1-dependent manner (Zheng et al., 2023). The ability of LbGp to combat oxidative stress and thus protect neuronal cells from oxidative stress and improve neuronal cell survival in patients with AD, however, remains unclear. *Lycium barbarum* has been proven to exert neuroprotective effects via multiple signaling pathways, including the PI3K/Akt/glycogen synthase kinase 3 beta, PI3K/Akt/mTOR, protein kinase C ε/nuclear factor erythroid 2-related factor 2 (Nrf2)/heme oxygenase-1 (HO-1), Kelch-like ECH-associated protein 1-Nrf2/HO-1, and NR2A and NR2B receptor-related signal transduction pathways (Huang et al., 2023). In addition, *Lycium barbarum*-derived nanovesicles have also been reported to promote cell proliferation and activity by activating the PI3K/Akt/mTOR/p70S6K signaling pathway (Xu et al., 2025). These findings suggest that the PI3K/Akt/p70S6K signaling pathway may be one of the mechanisms by which LbGp exerts its neuroprotective effect.

Neurotoxicity induced by OAβ is a key pathological feature driving neuronal damage in AD. This occurs primarily by triggering oxidative stress, impairing antioxidant defense systems, and disrupting neurotrophic signaling (Ionescu-Tucker and Cotman, 2021); however, the development of effective interventions that can specifically counteract Aβ-mediated oxidative damage and neurotoxicity remains an unresolved challenge. This study aimed to fill this gap by investigating the potential role of LbGp in countering Aβ-induced neurotoxicity and clarifying the antioxidant and neuroprotective mechanisms of LbGp, to provide experimental evidence for its potential as a therapeutic candidate for AD.

## Methods

### Preparation of *Lycium barbarum* glycopeptide

LbGp was provided by Ningxia Tianren Goji Biotechnology (Ningxia, China). LbGp was extracted from *Lycium barbarum* berries using an alcohol-free extraction method, as described previously (Kong et al., 2024). Briefly, purified LbGp, as a brown powder containing > 25.0% polysaccharides (w/w) and > 25.0% polymers, was dissolved in regular Dulbecco’s Modified Eagle Medium (DMEM; Gibco, Grand Island, NY, USA) at a concentration of 2000 mg/mL, shaken until fully dissolved, and then filtered through a 0.22 μM filter. Prior to experimentation, the LbGp was diluted to the target concentration for further study. The solution was stored at 4°C and used or replaced within 1 week.

### Preparation of oligomeric amyloid-beta1–42

Briefly, 1 mg of Aβ_42_ peptide (QYAOBIO ChinaPeptides Co., Ltd., Shanghai, China) was thawed on ice and then dissolved in cold 1,1,3,3,3-hexafluoro-2-propanol (HFIP) (Sigma, St. Louis, MO, USA) in a fume-hood environment, to obtain a 1 mM solution. The solution was incubated for 1 hour at room temperature to complete the Aβ monomerization process, and then transferred into two 1.5 mL Eppendorf tubes. The tubes were placed in a fume hood at room temperature and dried overnight to produce a transparent Aβ membrane. The membrane was resuspended in dimethyl sulfoxide to bring the concentration of Aβ_42_ to 5 mM, followed by the addition of high-glucose DMEM (Gibco) to produce a stock solution with a concentration of 100 μM. This stock solution was incubated overnight at 4°C and then centrifuged at 4°C and 17,000 × *g* for 10 minutes. The supernatant containing OAβ_1–42_ was collected and incubated with purified (azide-free) anti-Aβ antibody (BioLegend, San Diego, CA, USA, RRID: AB_2564634) at 4°C overnight for immunoblotting, to verify successful aggregation of OAβ_1–42_.

### Cell culture and treatment

Mouse Neuro 2a (N2a) cells (ATCC, Manassas, VA, USA, Cat# CCL-131; RRID: CVCL_0470) with immortalized characteristics were cultured in high-glucose DMEM (Thermo Fisher Scientific, Waltham, MA, USA) supplemented with 10% fetal bovine serum (FBS) (Invitrogen, Carlsbad, CA, USA). The cells were maintained in a humidified atmosphere containing 95% air and 5% carbon dioxide at a temperature of 37°C, with 1% penicillin-streptomycin mixture (Thermo Fisher Scientific) added to the culture medium to prevent contamination.

N2a cells were seeded in 96-well plates (1 × 10^4^ cells per well) or 12-well plates (1.5 × 10^5^ cells per well), and cultured in high-glucose DMEM supplemented with 1% FBS. To assess the dose-response cytotoxicity induced by OAβ_1–42_ or LbGp, the cells were treated for 22 hours with 1.25, 2.5, 5, or 10 mM OAβ_1–42_ (Merck Milli, Burlington, MA, USA), or 62.5, 125, 250, 500, or 1000 μg/mL LbGp for 24 hours, respectively, after determining the minimum toxic concentration of OAβ_1–42_. Cells were pretreated with LbGp (62.5, 125, 250, 500, and 1000 μg/mL) for 2 hours, followed by co-exposure to OAβ_1–42_ for 22 hours. To test if LbGp could mitigate the toxicity induced by OAβ_1–42_, the cells were divided into five groups for subsequent experiments. N2a cells were co-cultured with varying concentrations of Aβ_1–42_ for 22 hours, specifically assigned to the vehicle group and groups treated with 1.25, 2.5, 5, and 10 mM Aβ_1–42_, respectively. N2a cells were subsequently divided into a vehicle group and groups treated with 62.5, 125, 250, 500, and 1000 μg/mL LbGp for 24 hours. Finally, following pretreatment with different concentrations of LbGp for 2 hours, N2a cells were co-cultured with the lowest reliably toxic concentration of Aβ_1–42_ and LbGp, in the following groups: vehicle (control), Aβ + 62.5 μg/mL, Aβ + 125 μg/mL, Aβ + 250 μg/mL, Aβ + 500 μg/mL, and Aβ + 1000 μg/mL groups.

### Detection of mitochondrial metabolic activity (2,5-diphenyl-2H-tetrazolium bromide cytotoxicity assay)

We selected N2a cells for this study, based on their repeatability and ease of operation in previous reports (Wang et al., 2022; Wang and Jia, 2023). Mitochondrial metabolic activity was detected using a 2,5-diphenyl-2H-tetrazolium bromide (MTT) Kit, according to the manufacturer’s instructions (Beitian Biotechnology, Shanxi, China). N2a cells (5 × 10^3^) were seeded in 96-well plates. After 24 hours of culture and treatment, MTT solution (10 μL) was then added to each well and incubated for a further 4 hours, followed by the addition of 100 μL of formazan solution to each well, mixing, and incubation for another 2 hours. The cells were observed by light microscopy (Zeiss, Jena, Germany) until the formazan had completely dissolved, and the absorbance at 570 nm was then determined using a Multi-Mode Microplate Reader (Molecular Devices, San Jose, CA, USA).

### Cell viability assay

Cell viability was determined using a PrestoBlue assay, according to the manufacturer’s instructions (Thermo Fisher Scientific). PrestoBlue reagent was added to the N2a cells 4 hours before the end of the experiment and the cells were incubated at 37°C for 30 minutes. The absorbance was then measured at 570 nm using a Multi-Mode Microplate Reader (Molecular Devices) and the results were analyzed.

### Oxidative stress assay

N2a cells were treated with 5 μM CellROX^TM^ Green Reagent (Cat# C10444, Thermo Fisher Scientific) and incubated at 37°C for 30 minutes. DMEM medium was then removed from the four non-experimental groups and the cells were washed three times with phosphate-buffered saline (PBS) and fixed with 3.7% formaldehyde for 15 minutes. Finally, the cells underwent fluorescence imaging and analysis using the EVOS M7000 image system (Thermo Fisher Scientific) and ImageJ software (1.49 version, NIH, Bethesda, MD, USA).

### Immunocytochemistry staining

Stable N2a cells (2 × 10^4^ cells/well) were seeded in a 24-well plates with coverslips and pretreated with LbGp (1000 μg/mL) for 2 hours, followed by co-exposure to LbGp and OAβ_1–42_ for 22 hours and fixing in 4% paraformaldehyde (Merck, Darmstadt, Germany) for 10 minutes at room temperature. The cells were then washed three times with 1× PBS and permeabilized with 0.1% Triton X-100 (Merck) for 10 minutes at room temperature. After washing three times in PBS containing 0.1% Tween 20 (PBST) (BioBasic, Markham, Ontario, Canada) (5 minutes per wash), blocking solution containing 10% donkey serum (Sigma) was added to the cells, followed by incubation at 4°C for 2 hours. The cells were then incubated with anti-inducible nitric oxide synthase (iNOS) (rabbit, 1:500, CST, Danvers, MA, USA) and anti-Aβ antibody (mouse, 1:500, BioLegend) at 4°C in the dark overnight, washed three times in PBST, and treated with secondary antibodies Alexa Fluor^TM^ 488 donkey anti-rabbit (1:1000, Thermo Fisher Scientific) and Alexa Fluor^TM^ 568 donkey anti-mouse (1:1000, Thermo Fisher Scientific) for 2 hours in the dark at 4°C. The cells were washed three times in PBST and then incubated with 300 nM DAPI (Cat# D1306, Thermo Fisher Scientific) as a nuclear stain for 2 minutes. Fluorescence imaging was performed using the EVOS M7000 Imaging System (Thermo Fisher Scientific) and the results were analyzed using ImageJ software (1.49 version, NIH).

### Quantitative reverse transcription-polymerase chain reaction

N2a cells were pretreated with LbGp (1000 μg/mL) for 2 hours, followed by co-exposure to LbGp and OAβ_1–42_ for 6 hours. Total RNA was then extracted from N2a cell samples using an RNA extraction kit (Qiagen, Hilden, Germany, Cat# 74106) and reverse-transcribed into cDNA using a reverse transcription kit (Qiagen, Cat# 205413). The resultant cDNA samples were then analyzed by quantitative reverse transcription-polymerase chain reaction (qRT-PCR) using a SYBR Green PCR Kit (Qiagen, Cat# 208056). The amplification process consisted of an initial incubation at 95°C for 2 minutes, followed by 40 cycles of denaturation at 95°C for 5 seconds and annealing at 60°C for 15 seconds. Melting curve analysis was performed to ensure amplification specificity. The primer sequences for the tested target genes are presented in **Table 1**. The expression levels of the target genes were normalized to the housekeeping gene glyceraldehyde 3-phopshate dehydrogenase (*Gapdh*) and calculated using the 2^–ΔΔCT^ formula.

**Table 1:** Primer sequences used for quantitative reverse transcription-polymerase chain reaction

Gene	Forward (5'–3')	Reverse (5'–3')
*Sod1*	AAC CAG TTG TGT TGT CAG GAC	CCA CCA TGT TTC TTA GAG TGA GG
*Sod2*	CAG ACC TGC CTT ACG ACT ATG G	CTC GGT GGC GTT GAG ATT GTT
*Gpx4*	TGT GCA TCC CGC GAT GAT T	CCC TGT ACT TAT CCA GGC AGA
*Pi3k*	ACA CCA CGG TTT GGA CTA TGG	GGC TAC AGT AGT GGG CTT GG
*Akt*	ATG AAC GAC GTA GCC ATT GTG	TTG TAG CCA ATA AAG GTG CCA T
*p70S6k*	GGG GCT ATG GAA AGG TTT TTC A	CTT TAG TCA GCG ACA GAA GGA C
*Gapdh*	AGG TCG GTG TGA ACG GAT TTG	GGG GTC GTT GAT GGC AAC A

Gapdh: Glyceraldehyde 3-phopshate dehydrogenase; Pi3k: phosphoinositide 3-kinase; SOD: superoxide dismutase.

### Western blot assay

N2a cells were pretreated with LbGp (1000 μg/mL) for 2 hours followed by co-exposure to OAβ_1–42_ for 22 hours, and then lysed on ice using RIPA buffer (BioBasic), according to the manufacturer’s protocol. The lysate was centrifuged at 16,000 × *g* for 20 minutes, and the supernatant was collected and stored at –80°C. The protein concentration was measured by Pierce^TM^ BCA Protein Assay Kit (Thermo Fisher Scientific). Total proteins were subjected to sodium dodecyl sulfate-polyacrylamide gel electrophoresis on a 10% polyacrylamide gel and transferred to a polyvinylidene difluoride membrane (Thermo Fisher Scientific). The membranes were blocked with 5% non-fat milk (Sigma) diluted in PBST (BioBasic) at 4°C for 2 hours and then incubated with primary antibodies against β-actin (rabbit, 1:1000, CST), superoxide dismutase 1 (SOD1; rabbit, 1:1000, CST), SOD2 (rabbit, 1:1000, CST), brain-derived neurotrophic factor (BDNF; rabbit, 1:1000, CST), PI3K (mouse, 1:1000, Invitrogen), Akt (rabbit, 1:1000, Invitrogen), and p70S6K (rabbit, 1:1000, CST) overnight at 4°C. After three washes with PBST, they were then incubated for 1 hour with either anti-mouse IgG-horseradish peroxidase (HRP) (donkey, 1:2000, Invitrogen) or anti-rabbit IgG-HRP (donkey, 1:2000, Invitrogen). Finally, the blots were detected using an enhanced chemiluminescent reagent (R&D Systems, Minneapolis, MN, USA). The grayscale values were analyzed using ImageJ software.

### Statistical analysis

Graphs were created using GraphPad Prism 8.0 software (GraphPad Software, San Diego, CA, USA) and data analysis was conducted using SPSS for Windows (Version 20.0; IBM, Armonk, NY, USA). Differences among multiple groups were analyzed by one-way analysis of variance, followed by Fisher’s least significant difference test, or Dunnett’s test when variances were not equal among all groups. Differences between two groups were analyzed by two-tailed Student’s *t*-tests. Data were expressed as mean ± standard deviation, and *P* < 0.05 was considered statistically significant.

## Results

### *Lycium barbarum* glycopeptide reduces the cytotoxicity of oligomeric amyloid-beta1–42

**Figure 1:**
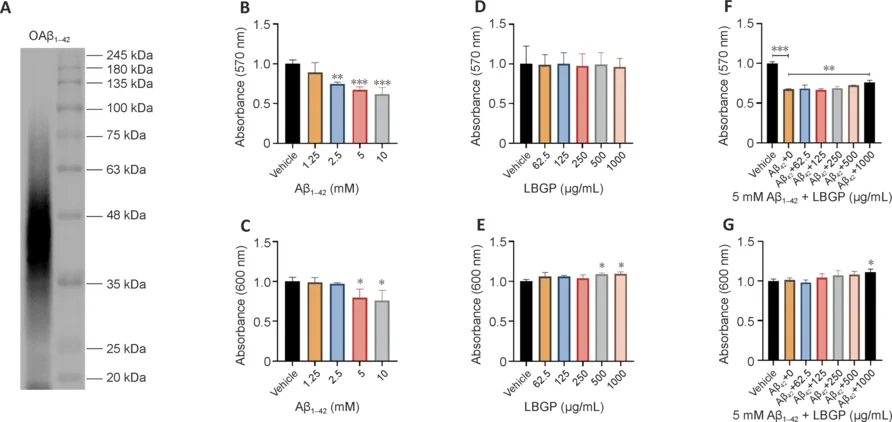
Detection of OAβ_1–42_ and N2a cytotoxicity. (A) Immunoblotting of OAβ_1–42_. (B) MTT cytotoxicity assay in N2a cells stimulated with different concentrations of OAβ_1–42_. (C) PrestoBlue cell viability assay of N2a cells stimulated with different concentrations of OAβ_1–42_. (D) MTT cytotoxicity assay of N2a cells stimulated with different concentrations of LbGp. (E) PrestoBlue cell viability assay of N2a cells stimulated with different concentrations of LbGp. (F) MTT cytotoxicity assay conducted after LbGp treatment for 2 hours followed by co-exposure to LbGp and 5 mM OAβ_1–42_ for 22 hours to stimulate N2a cells. (G) PrestoBlue cell viability assay performed after LbGp treatment for 2 hours followed by co-exposure to LbGp and 5 mM OAβ_1–42_ for 22 hours to stimulate N2a cells. Data are expressed as mean ± SD (*n* = 3, *P < 0.05, **P < 0.01, ***P < 0.001). Aβ: Amyloid-beta; LbGp: *Lycium barbarum* glycopeptide; MTT: 2,5-diphenyl-2H-tetrazolium bromide; OAβ: oligomeric amyloid-beta.

We verified the successful preparation of OAβ_1–42_ and analyzed the differences in cytotoxicity among the different treatment groups. Following the above experimental protocol, western blot protein bands were developed after dissolving Aβ_1–42_ peptide in HFIP and re-suspending in dimethyl sulfoxide. The demonstrated molecular weight (MW) of Aβ_1–42_ protein was approximately 35–50 kDa (**Figure 1A**), which was consistent with the MW of OAβ_1–42_ in previous experiments (Prakash et al., 2019). OAβ_1–42_ demonstrated dose-dependent cytotoxicity in N2a cells after culture for 22 hours, as detected by MTT assay. Co-culture of N2a cells with different concentrations of Aβ_1–42_ (1.25, 2.5, 5, 10 mM) decreased the MTT assay substrate to 0.75 ± 0.03 (*P* < 0.01), 0.68 ± 0.04 (*P* < 0.001), and 0.62 ± 0.08 times (*P* < 0.001) in the 2.5, 5, and 10 mM groups, respectively, compared with the vehicle group (**Figure 1B**). We confirmed the experimental results in N2a cells using the PrestoBlue test, and showed that the PrestoBlue assay substrate decreased to 0.8 ± 0.11 (*P* < 0.05) and 0.76 ± 0.13 times (*P* < 0.05) in the 5 and 10 mM groups, respectively, compared with the vehicle group (**Figure 1C**). For the reliability of the experiment, we choose 5 mM as the effective concentration for N2a cell damage stimulation in subsequent experiments. The MTT cytotoxicity assay showed no significant difference among N2a cells stimulated with 62.5, 125, 250, 500, and 1000 μg/mL LbGp (**Figure 1D**), indicating that LbGp had no significant cytotoxicity in N2a cells. Similarly, the PrestoBlue test showed slight increases at 500 and 1000 μg/mL of 1.1 ± 0.014 (*P* < 0.05) and 1.1 ± 0.024 times (*P* < 0.05), respectively, compared with the vehicle group, suggesting that these concentrations of LbGp may promote the activity or proliferation of N2a cells (Luzak et al., 2022; **Figure 1E**). We stimulated N2a cells with 5 mM OAβ_1–42_, followed by different concentrations of LbGp for 24 hours. MTT assay results showed that OAβ_1–42_ decreased the MTT substrate to 0.67 ± 0.0055 times compared with the vehicle group (*P* < 0.001). Meanwhile, pretreatment with 1000 μg/mL LbGp for 2 hours followed by co-exposure to LbGp and OAβ_1–42_ for 22 hours, effectively counteracted the cytotoxicity of OAβ_1–42_ (*P* < 0.01; **Figure 1F**). Under the same conditions, treatment with 1000 μg/mL LbGp increased the PrestoBlue assay substrate by 1.1 ± 0.041 times compared with the vehicle group (*P* < 0.05), which could enhance the vitality of N2a cells or promote cell proliferation (**Figure 1G**). These results demonstrated the successful preparation of OAβ_1–42_, and showed that 1000 μg/mL LbGp could reduce the cytotoxic reaction of OAβ_1–42_.

### *Lycium barbarum* glycopeptide alleviates reactive oxygen species stress caused by oligomeric amyloid-beta1–42

**Figure 2:**
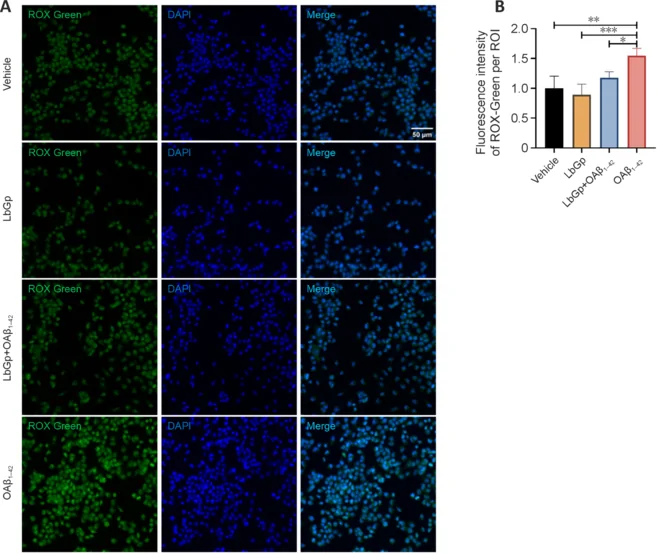
Qualitative and quantitative detection of reactive oxygen species in N2a cells in the experimental groups. (A) Cells in the different experimental groups were stained with CellROX™ Green Reagent (scale bar: 50 μm). (B) ROX Green fluorescence intensity analysis. Data are expressed as mean ± SD (*n* = 4, *P < 0.05, **P < 0.01, ***P < 0.001). LbGp: *Lycium barbarum* glycopeptide; OAβ: oligomeric amyloid-beta; ROI: region of interest.

We compared the cellular ROS contents among the different treatment groups. N2a cells were pretreated with LbGp (1000 μg/mL) for 2 hours, followed by co-exposure to LbGp and OAβ_1–42_ for 22 hours, and cells from each group were stained using CellROX^TM^ Green Reagent. ROX expression was significantly increased in the OAβ_1–42_ group, whereas cells in the LbGp and vehicle control groups showed no excessive ROX expression. Moreover, co-treatment with LbGp and OAβ_1–42_ significantly reduced ROX expression compared with the OAβ_1–42_ alone group (**Figure 2A**). The relative fluorescence intensities in the LbGp + OAβ_1–42_ and OAβ_1–42_ groups were 1.2 ± 0.098 and 1.5 ± 0.12, respectively, indicating a significant reduction in the LbGp group (*P* < 0.05; **Figure 2B**). These findings suggest that treatment with 1 mg/mL LbGp effectively mitigated the oxidative stress response induced by OAβ_1–42_.

### *Lycium barbarum* glycopeptide reduces intracellular deposition of oligomeric amyloid-beta1–42 and inhibits oligomeric amyloid-beta1–42-induced inducible nitric oxide synthase expression

**Figure 3:**
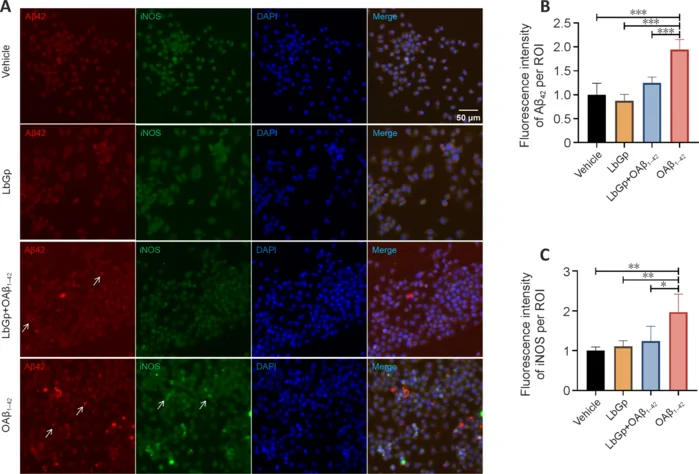
Immunofluorescence staining of N2a cells in the experimental groups. (A) Staining and localization of Aβ_42_ protein (arrows), iNOS (arrows), and N2a cell nuclei (DAPI) (scale bar: 50 μm). (B, C) Aβ_42_ (B) and iNOS (C) fluorescence intensity analysis. Data are expressed as mean ± SD (*n* = 4, *P < 0.05, **P < 0.01, ***P < 0.001). DAPI: 4′,6-Diamidino-2-phenylindole; iNOS: inducible nitric oxide synthase; LbGp: *Lycium barbarum* glycopeptide; OAβ: oligomeric amyloid-beta; ROI: region of interest.

We compared the deposition of cellular OAβ_1–42_ among the different treatment groups and analyzed iNOS by quantitative immunofluorescence. We examined the localizations of OAβ_1–42_ protein and iNOS in N2a cells across different experimental groups using immunofluorescence staining. Intracellular precipitation of OAβ_1–42_ protein and expression of iNOS, as an inflammatory cytokine induced by OAβ_1–42_, were both significantly reduced in the LbGp + OAβ_1–42_ group compared with the OAβ_1–42_ group; however, there was no significant difference in iNOS expression between the LbGp + OAβ_1–42_ and LbGp groups (**Figure 3A**). The relative fluorescence intensities of OAβ_1–42_ in the LbGp + OAβ_1–42_ and OAβ_1–42_ groups were 1.2 ± 0.12 and 1.9 ± 0.21, respectively, indicating a significant reduction in OAβ_1–42_ precipitation in the LbGp + OAβ_1–42_ group (*P* < 0.001; **Figure 3B**). Additionally, fluorescence intensity analysis demonstrated a significant down-regulation in iNOS expression in the LbGp + OAβ_1–42_ group (*P* < 0.05), with relative fluorescence intensities for the LbGp + OAβ_1–42_ and OAβ_1–42_ groups of 1.2 ± 0.38 and 2.0 ± 0.45, respectively (**Figure 3C**). These results indicate that LbGp reduced the intracellular deposition of OAβ_1–42_ and inhibited the expression of iNOS caused by OAβ_1–42_.

### *Lycium barbarum* glycopeptide counteracts the down-regulation of antioxidant enzyme genes and Pi3k-Akt-p70S6k signal genes caused by oligomeric amyloid-beta1–42

**Figure 4:**
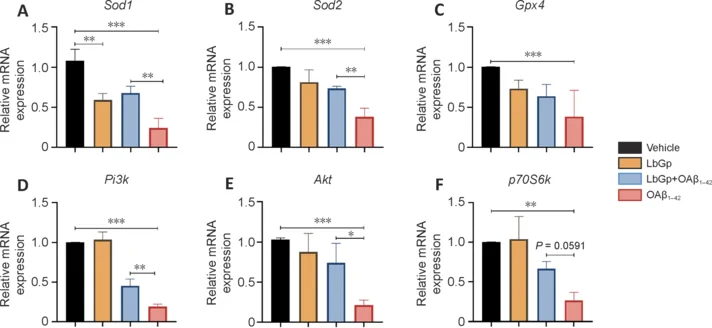
Gene expression levels in the experimental groups after treating N2a cells with LbGp for 8 hours. (A–C) Oxidative stress-related genes: *Sod1* (A), *Sod2* (B), and *Gpx4* (C). (D–F) Pi3k signaling pathway genes: *Pi3k* (D), *Akt* (E), *p70S6k* (F). Relative expression levels. Data are expressed as mean ± SD (*n* = 3, *P < 0.05, **P < 0.01, ***P < 0.001). LbGp: *Lycium barbarum* glycopeptide; OAβ: oligomeric amyloid-beta; Pi3k: phosphoinositide 3-kinase; sod: superoxide dismutase.

We compared the expression levels of antioxidant enzymes and genes related to the Pi3k-Akt-p70S6k pathway among the treatment groups. We further examined the expression of antioxidant enzymes in the different experimental groups, including expression levels of *Sod1*, *Sod2*, and *Gpx4* genes. Sod1 expression was significantly up-regulated in the LbGp + OAβ_1–42_ group (relative expression 0.68 ± 0.085) compared with the OAβ_1–42_ group (relative expression 0.24 ± 0.12; *P* < 0.01). Interestingly, the relative expression level of Sod1 in the LbGp group (0.59 ± 0.082) was lower than that in the vehicle group (*P* < 0.01). Sod1 expression was slightly down-regulated in the LbGp group compared with the LbGp + OAβ group, but the difference was not significant (**Figure 4A**). Sod2 gene expression was significantly higher in the LbGp + OAβ_1–42_ group compared with the OAβ_1–42_ group (*P* < 0.01; **Figure 4B**). In contrast, Gpx4 expression was reduced to 0.38 ± 0.33 times in the OAβ_1–42_ group compared with the vehicle group, with no significant difference compared with the OAβ_1–42_ group (**Figure 4C**). Furthermore, relative expression of the Pi3k gene increased significantly from 0.19 ± 0.032 in the OAβ_1–42_ group to 0.45 ± 0.085 in the LbGp + OAβ_1–42_ group (*P* < 0.05; **Figure 4D**), and expression of the downstream Akt gene increased significantly from 0.21 ± 0.063 to 0.74 ± 0.24 (*P* < 0.05; **Figure 4E**). Additionally, relative expression levels of the downstream gene p70S6k in the OAβ_1–42_ and LbGp + OAβ_1–42_ groups were 0.27 ± 0.10 and 0.67 ± 0.093, respectively (*P* = 0.0591; **Figure 4F**). These results indicated that LbGp counteracted the down-regulation of antioxidant enzyme genes (*Sod1*, *Sod2*, and *Gpx4*) and Pi3k-Akt-p70S6k signal genes caused by OAβ_1–42_.

### *Lycium barbarum* glycopeptide counteracts down-regulation of antioxidant enzymes and brain-derived neurotrophic factor caused by oligomeric amyloid-beta1–42

**Figure 5:**
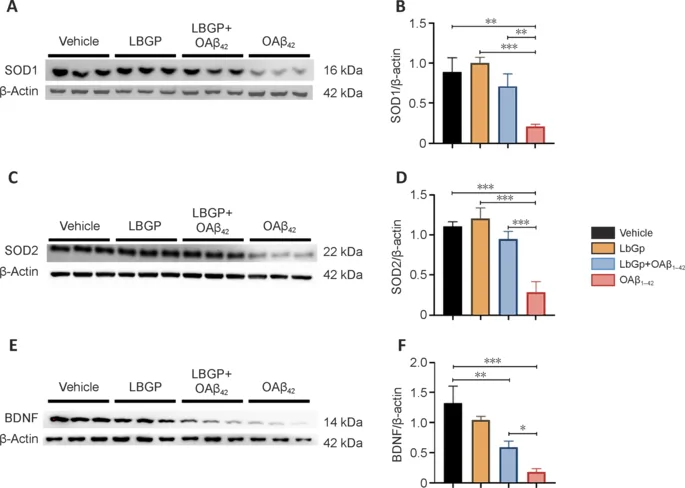
Protein expression levels in the experimental groups after treating N2a cells with LbGp. (A–F) Representative western blot bands (A, C, E) and relative protein expression (B, D, F) of SOD1, SOD2, and BDNF. Data are expressed as mean ± SD (*n* = 3, *P < 0.05, **P < 0.01, ***P < 0.001). BDNF: Brain-derived neurotrophic factor; LbGp: *Lycium barbarum* glycopeptide; OAβ: oligomeric amyloid-beta; SOD: superoxide dismutase.

We compared antioxidant enzyme and BDNF protein levels among the different treatment groups by western blot, with β-actin as an internal reference for protein quantification correction. SOD1 protein levels were significantly up-regulated in the LbGp + OAβ_1–42_ group compared with the OAβ_1–42_ group. Protein grayscale analysis showed that relative SOD1/β-actin values in the OAβ_1–42_ and LbGp + OAβ_1–42_ groups of 0.21 ± 0.029 and 0.71 ± 0.15, respectively (*P* < 0.01; **Figure 5A** and **B**). Similarly, SOD2 protein levels were significantly up-regulated in the LbGp + OAβ_1–42_ group compared with the OAβ_1–42_ group, and ImageJ protein grayscale analysis showed relative SOD2/β-actin values of 0.28 ± 0.14 and 0.94 ± 0.10 in the OAβ_1–42_ and LbGp + OAβ_1–42_ groups, respectively (*P* < 0.05; **Figure 5C** and **D**). BDNF protein levels were significantly decreased in the OAβ_1–42_ group, but LbGp up-regulated the expression of BDNF protein in the LbGp + OAβ_1–42_ group, with relative BDNF/β-actin values of 0.18 ± 0.058 and 0.59 ± 0.10 in the OAβ_1–42_ and LbGp + OAβ_1–42_ groups, respectively (**Figure 5E** and **F**). These results demonstrated that LbGp counteracted the down-regulation of antioxidant enzymes (SOD1, SOD2) and BDNF caused by OAβ_1–42_.

### *Lycium barbarum* glycopeptide counteracts down-regulation of PI3K/Akt/p70S6K pathway proteins caused by oligomeric amyloid-beta1–42

**Figure 6:**
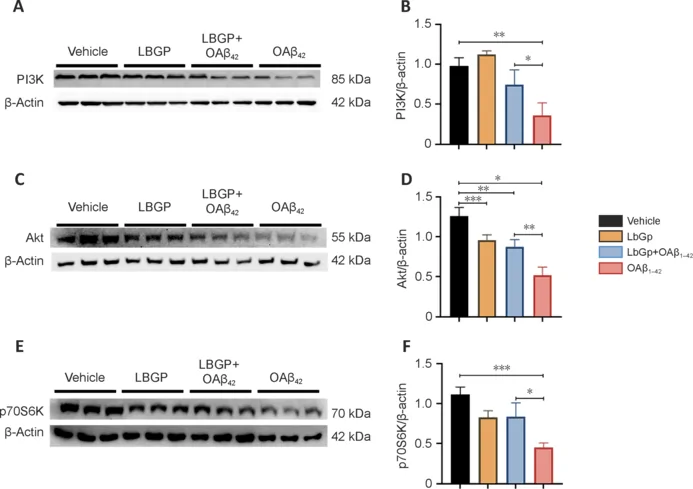
PI3K and Akt protein expression levels in the experimental groups after treating N2a cells with LbGp. (A–F) Representative western blot bands (A, C, E) and relative protein expression (B, D, F) of PI3K, Akt, and p70S6K. Data are expressed as mean ± SD (*n* = 3, *P < 0.05, **P < 0.01, ***P < 0.001). LbGp: *Lycium barbarum* glycopeptide; OAβ: oligomeric amyloid-beta; PI3K: phosphoinositide 3-kinase.

We compared the expression levels of antioxidant PI3K/Akt/p70S6K pathway proteins among the different treatment groups by western blot, with β-actin as an internal reference for quantification correction. PI3K/Akt/p70S6K pathway proteins were significantly up-regulated in the LbGp + OAβ_1–42_ group compared with the OAβ_1–42_ group, and ImageJ protein grayscale analysis indicated relative PI3k/β-actin values of 0.36 ± 0.19 and 0.74 ± 0.16 in the OAβ_1–42_ and LbGp + OAβ_1–42_ groups, respectively (*P* < 0.05; **Figures 6A** and **B**). Akt protein levels were also significantly up-regulated in the LbGp + OAβ_1–42_ group compared with the OAβ_1–42_ group, with relative Akt/β-actin values of 0.52 ± 0.11 and 0.87 ± 0.095 in the OAβ_1–42_ and LbGp + OAβ_1–42_ groups, respectively (*P* < 0.01; **Figure 6C** and **D**). Similarly, p70S6K protein levels were significantly up-regulated in the LbGp + OAβ_1–42_ group compared with the OAβ_1–42_ group, with relative p70S6K/β-actin values of 0.45 ± 0.054 and 0.83 ± 0.17 in the OAβ_1–42_ and LbGp + OAβ_1–42_ groups, respectively (*P* < 0.05; **Figure 6E** and **F**). These results indicated that LbGp counteracted the down-regulation of PI3K/Akt/p70S6K pathway proteins caused by OAβ_1–42_.

## Discussion

Oxidative stress plays a crucial role in the onset and progression of many diseases, and given the high oxygen consumption by the brain, oxidative stress is particularly prominent in degenerative neurological diseases of the central nervous system, such as AD, Parkinson’s disease, Huntington’s disease, and amyotrophic lateral sclerosis (Zhang et al., 2014). Aβ is a critical pathogenic factor in AD and has a strong association with oxidative stress damage. Elevated levels of Aβ_1–40_ and Aβ_1–42_ have been associated with increased levels of protein and lipid oxidation products in the hippocampus in AD (Butterfield and Lauderback, 2002), including F2-isoprostane-α (cerebrospinal fluid, frontal and temporal lobes) (Montine et al., 1999), acrolein (amygdala and hippocampus/para-hippocampal gyrus) (Lovell et al., 2001), and hydroxynonenal (frontal cortex) (Ansari and Scheff, 2010). Notably, stimulation with low concentrations of ROS can protect neurons from subsequent damage. Pennisi et al. (2017) elaborated on this phenomenon and attributed this characteristic to hormesis. Extensive research has demonstrated activation of the NF-κB signaling pathway (*IκBα* and *NF-κB p65*) in oxidative stress injury, as well as inhibition of nuclear factor erythroid-2-related factor 2 (Nrf2)/heme oxygenase 1 (Ho-1) signaling (Han et al., 2022), which in turn affects the expression of genes including *Ho-1*, *Sod*, and *Gpx* (Le et al., 2024).

LbGp extracted from *Lycium barbarum* is considered to be the main active therapeutic component of this plant, primarily associated with its impressive antioxidant effects (Dai et al., 2023; Ma et al., 2025). LbGp is a peptidoglycan with a short peptide backbone and complex branched polysaccharide parts. In our experiments, even the highest concentration of LbGp (1000 μg/mL) showed no cytotoxicity in N2a cells, while only the highest concentration had a therapeutic effect against OAβ_1–42_ toxicity. Previous reports indicated that one of the active components of LbGp, LbGp5 (100 μg/mL), had the strongest antioxidant activity (Huang et al., 2001) and could promote the survival of human fibroblasts and improve the viability of full-thickness human skin and dermal fibroblasts (Zhao et al., 2005). LbGp has also been found to inhibit the growth of various cancer cells through cell cycle arrest and apoptosis inhibition. LbGp (800 μg/mL) adjusted the cell cycle G0/G1 phase block in liver cancer cells (SMMC-7721 and HepG2), cervical cancer cells (HeLa), gastric cancer cells (SGC-7901), and human breast cancer cells (MCF-7), as well as altering mitochondrial function, activating oxidative stress, and regulating the MAPK signaling pathway to induce apoptosis, with no effect on normal liver cells (Gong et al., 2020). *In vivo* experimental studies have also demonstrated that oral administration of LbGp at a dose of 100 mg/kg for 12 weeks can safely and effectively reverse aging phenotypes in mice (Peng et al., 2025). Furthermore, a 3-week oral administration at 10 mg/kg was similarly found to modulate the composition and diversity of the gut microbiota, ultimately preventing acute colitis by alleviating inflammation and oxidative stress (Huang et al., 2022). This suggests that LbGp has a wide effective drug concentration range and is a potential functional food ingredient that is non-toxic to normal cells *in vitro*. The core advantage of LbGp lies in its natural safety profile and its ability to precisely modulate the microenvironment for neuronal survival. By synergistically reducing oxidative stress and promoting cell viability, LbGp not only rapidly alleviates cellular toxicity but also holds long-term potential for enhancing neuronal survival. These properties position it as a high-value candidate for the development of functional foods and therapeutics targeting Alzheimer’s disease.

OAβ_1–42_-induced ROS production in N2a cells caused significant green fluorescence using CellROX™ Green Reagent, consistent with previous reports (Qiao et al., 2024); however, LbGp significantly reduced this fluorescence, indicating that LbGp effectively alleviated the increase in ROS caused by OAβ_1–42_ stimulation. This may be one of the reasons for the increased cell activity and reduced cytotoxicity. There is a close relationship between oxidative stress, neuroinflammation, and Aβ production (Cheignon et al., 2018). Additionally, OAβ can directly induce oxidative stress, promoting cell damage and apoptosis (Urrutia et al., 2017; Yang et al., 2017). We previously showed that *Lycium barbarum* extract significantly improved the symptoms of AD in mice by regulating the state of the microglia and reducing inflammation in the central nervous system (Sun et al., 2024). As the main active component of this extract, LbGp may also have some anti-inflammatory effects. In addition, we also reported that LbGp reversed the reduction in antioxidant enzymes (SOD1 and PRDX5) in microglia caused by H_2_O_2_ (Zheng et al., 2022). LbGp has also demonstrated the ability to inhibit oxidative stress and inflammation by activating the Sirt3 signaling pathway, and significantly improved nerve damage caused by cerebral hemorrhage (Ma et al., 2025). LbGp relieved oxidative stress and lipid peroxidation in the medial prefrontal cortex in a chronic stimulation anxiety mouse model by inhibiting the ferroptosis pathway, showing strong neuroprotective ability (Dai et al., 2023). LbGp can also improve the inflammatory microenvironment by inhibiting the NF-κB and pyroptosis pathways, and inducing microglia to secrete docosahexaenoic acid, which in turn inhibits neuroinflammation via the MAPK/NF-κB and pyroptosis pathways (Jiang et al., 2023). The current results showed that treatment with LbGp and the addition of Aβ_1–42_ reduced the deposition of Aβ in N2a cells, and LbGp also decreased expression of the inflammation-related protein iNOS. This may be one of the reasons why LbGp alleviates the cytotoxicity of OAβ; however, further research is needed to determine the specific mechanism and to clarify the anti-inflammatory effects of LbGp in non-immune cell populations, such as N2a.

Peroxisomes play an important role in protecting against ROS. Previous research showed that expression levels of peroxisomal (peroxisomal membrane protein 70, catalase, alternative oxidase, and thyolase) and peroxisome-related proteins (peroxisomal targeting sequence type 1 receptor, GPX, and SOD) in the hippocampal region were significantly reduced in the early stages of AD in mice, indicating that oxidative stress damage is already present in the early stages of AD onset (Cimini et al., 2009). The present results showed that LbGp could effectively increase the expression of the antioxidant enzymes SOD1, SOD2, and GPX4, which may also be an important mechanism by which LbGp eliminates ROS, improves mitochondrial activity in N2a cells, and enhances cell vitality.

The stimulation of newborn neurons by Aβ can lead to the down-regulation of the PI3K/Akt/mTOR/p70S6K signaling pathway, stopping the growth of axons and dendrites, ultimately leading to their death (Li et al., 2010). This demonstrates that this signaling pathway plays a crucial role in neuronal survival and synaptic plasticity in the progression of AD. The current experimental results are in accord with previous reports, showing that OAβ can significantly reduce the PI3K/Akt/p70S6K signaling pathway in N2a cells, and this trend can be reversed by the addition of LbGp. BDNF expression was also enhanced, indicating that LbGp can effectively up-regulate the PI3K/Akt/p70S6K signaling pathway and promote the expression of BDNF, thus playing a crucial role in improving cell survival and synaptic plasticity. Nevertheless, this study merely detected the protein expression of BDNF, and further experiments are needed to determine if its synthesis and degradation pathways, along with key regulatory points, are regulated by the PI3K/Akt/p70S6K signaling pathway.

This study had certain limitations. First, the experiments were only carried out employing the N2a cell model, and the *in vivo* efficacy of LbGp and its blood–brain barrier permeability were not verified *in vivo* in AD transgenic animal models. The overall therapeutic effect at the organismal level is thus yet to be established. Second, the direct molecular targets of LbGp, such as PI3K and antioxidant enzymes, have not been clearly determined, and further studies are needed to identify the binding mechanisms. Third, the differential impacts of LbGp across diverse pathological stages of AD have not been examined, and the optimal therapeutic window of LbGp is thus undefined.

In conclusion, the current study demonstrates that LbGp 1000 μg/mL is capable of alleviating the cytotoxicity and enhancing the viability of N2a cells induced by OAβ_1–42_. LbGp can mitigate the OAβ_1–42_-induced elevations of ROS and the inflammatory factor iNOS, up-regulate the mRNA expression of antioxidative enzymes (SOD1, SOD2), and activate the PI3K/Akt/p70S6K pathway, thereby promoting the expression of BDNF to exert neuroprotective effects. These findings suggest that LbGp may be an effective candidate drug for the treatment of AD.

## Data Availability

*No additional data are available.*

## References

[R1] Ansari MA, Scheff SW (2010). Oxidative stress in the progression of Alzheimer disease in the frontal cortex. J Neuropathol Exp Neurol.

[R2] Bai R, Guo J, Ye XY, Xie Y, Xie T (2022). Oxidative stress: The core pathogenesis and mechanism of Alzheimer’s disease. Ageing Res Rev.

[R3] Browne D, McGuinness B, Woodside JV, McKay GJ (2019). Vitamin E and Alzheimer’s disease: what do we know so far?. Clin Interv Aging.

[R4] Butterfield DA, Lauderback CM (2002). Lipid peroxidation and protein oxidation in Alzheimer’s disease brain: potential causes and consequences involving amyloid beta-peptide-associated free radical oxidative stress. Free Radic Biol Med.

[R5] Cheignon C, Tomas M, Bonnefont-Rousselot D, Faller P, Hureau C, Collin F (2018). Oxidative stress and the amyloid beta peptide in Alzheimer’s disease. Redox Biol.

[R6] Chen LL, Fan YG, Zhao LX, Zhang Q, Wang ZY (2023). The metal ion hypothesis of Alzheimer’s disease and the anti-neuroinflammatory effect of metal chelators. Bioorg Chem.

[R7] Choi EH, Kim MH, Park SJ (2024). Targeting mitochondrial dysfunction and reactive oxygen species for neurodegenerative disease treatment. Int J Mol Sci.

[R8] Cimini A, Moreno S, D’Amelio M, Cristiano L, D’Angelo B, Falone S, Benedetti E, Carrara P, Fanelli F, Cecconi F, Amicarelli F, Cerù MP (2009). Early biochemical and morphological modifications in the brain of a transgenic mouse model of Alzheimer’s disease: a role for peroxisomes. J Alzheimers Dis.

[R9] Dai Y, Guo J, Zhang B, Chen J, Ou H, He RR, So KF, Zhang L (2023). Lycium barbarum (Wolfberry) glycopeptide prevents stress-induced anxiety disorders by regulating oxidative stress and ferroptosis in the medial prefrontal cortex. Phytomedicine.

[R10] Dinkova-Kostova AT, Kostov RV, Kazantsev AG (2018). The role of Nrf2 signaling in counteracting neurodegenerative diseases. FEBS J.

[R11] Fu YW, Peng YF, Huang XD, Yang Y, Huang L, Xi Y, Hu ZF, Lin S, So KF, Ren CR (2021). Lycium barbarum polysaccharide-glycoprotein preventative treatment ameliorates aversive. Neural Regen Res.

[R12] Ganguly U, Kaur U, Chakrabarti SS, Sharma P, Agrawal BK, Saso L, Chakrabarti S (2021). Oxidative stress, neuroinflammation, and NADPH oxidase: implications in the pathogenesis and treatment of Alzheimer’s disease. Oxid Med Cell Longev.

[R13] Gao W, Chen R, Xie N, Tang D, Zhou B, Wang D (2020). Duloxetine-induced neural cell death and promoted neurite outgrowth in N2a cells. Neurotox Res.

[R14] Gong G, Liu Q, Deng Y, Dang T, Dai W, Liu T, Liu Y, Sun J, Wang L, Liu Y, Sun T, Song S, Wang Z, Huang L (2020). Arabinogalactan derived from Lycium barbarum fruit inhibits cancer cell growth via cell cycle arrest and apoptosis. Int J Biol Macromol.

[R15] Han EJ, Zhang C, Kim HS, Kim JY, Park SM, Jung WK, Ahn G, Cha SH (2022). Sargachromenol isolated from sargassum horneri attenuates glutamate-induced neuronal cell death and oxidative stress through inhibition of MAPK/NF-κB and activation of Nrf2/HO-1 signaling pathway. Mar Drugs.

[R16] Huang LJ, Tian GY, Wang ZF, Dong JB, Wu MP (2001). Studies on the glycoconjugates and glycans from Lycium barbarum L in inhibiting low density lipoprotein (LDL) peroxidation. Yao Xue Xue Bao.

[R17] Huang Y, Zheng Y, Yang F, Feng Y, Xu K, Wu J, Qu S, Yu Z, Fan F, Huang L, Qin M, He Z, Nie K, So KF (2022). Lycium barbarum Glycopeptide prevents the development and progression of acute colitis by regulating the composition and diversity of the gut microbiota in mice. Front Cell Infect Microbiol.

[R18] Huang Y, Zhang X, Chen L, Ren BX, Tang FR (2023). Lycium barbarum ameliorates neural damage induced by experimental ischemic stroke and radiation exposure. Front Biosci (Landmark Ed).

[R19] Ionescu-Tucker A, Cotman CW (2021). Emerging roles of oxidative stress in brain aging and Alzheimer’s disease. Neurobiol Aging.

[R20] Jiang C, Chen Z, Xiong H, Yang X, Liao W, Chen G, Huang C, Zhu G, Yu H, Ma L (2024). Lycium barbarum berry extract improves female fertility against aging-related oxidative stress in the ovary. Food Funct.

[R21] Jiang Z, Zeng Z, He H, Li M, Lan Y, Hui J, Bie P, Chen Y, Liu H, Fan H, Xia H (2023). Lycium barbarum glycopeptide alleviates neuroinflammation in spinal cord injury via modulating docosahexaenoic acid to inhibiting MAPKs/NF-kB and pyroptosis pathways. J Transl Med.

[R22] Kepp KP, Robakis NK, Høilund-Carlsen PF, Sensi SL, Vissel B (2023). The amyloid cascade hypothesis: an updated critical review. Brain.

[R23] Kong Q, Han X, Cheng H, Liu J, Zhang H, Dong T, Chen J, So KF, Mi X, Xu Y, Tang S (2024). Lycium barbarum glycopeptide (wolfberry extract) slows N-methyl-N-nitrosourea-induced degradation of photoreceptors. Neural Regen Res.

[R24] Kwon CH, Zhu X, Zhang J, Baker SJ (2003). mTor is required for hypertrophy of Pten-deficient neuronal soma in vivo. Proc Natl Acad Sci U S A.

[R25] Lafay-Chebassier C, Paccalin M, Page G, Barc-Pain S, Perault-Pochat MC, Gil R, Pradier L, Hugon J (2005). mTOR/p70S6k signalling alteration by Abeta exposure as well as in APP-PS1 transgenic models and in patients with Alzheimer’s disease. J Neurochem.

[R26] Le MPT, Marasinghe CK, Je JY (2024). Chitosan oligosaccharides: A potential therapeutic agent for inhibiting foam cell formation in atherosclerosis. Int J Biol Macromol.

[R27] Li L, Xu B, Zhu Y, Chen L, Sokabe M, Chen L (2010). DHEA prevents Aβ25–35-impaired survival of newborn neurons in the dentate gyrus through a modulation of PI3K-Akt-mTOR signaling. Neuropharmacology.

[R28] Lovell MA, Xie C, Markesbery WR (2001). Acrolein is increased in Alzheimer’s disease brain and is toxic to primary hippocampal cultures. Neurobiol Aging.

[R29] Luzak B, Siarkiewicz P, Boncler M (2022). An evaluation of a new high-sensitivity PrestoBlue assay for measuring cell viability and drug cytotoxicity using EA.hy926 endothelial cells. Toxicol In Vitro.

[R30] Ma CS, Han B, Meng SC, Bai M, Yi WJ, Zhang LY, Duan MY, Wang YJ, He MT (2025). Lycium barbarum glycopeptide attenuates intracerebral hemorrhage-induced inflammation and oxidative stress via activation of the Sirt3 signaling pathway. Int Immunopharmacol.

[R31] Marvi F, Chen YH, Sawan M (2025). Alzheimer’s disease diagnosis in the preclinical stage: normal aging or dementia. IEEE Rev Biomed Eng.

[R32] Montine TJ, Beal MF, Cudkowicz ME, O’Donnell H, Margolin RA, McFarland L, Bachrach AF, Zackert WE, Roberts LJ, Morrow JD (1999). Increased CSF F2-isoprostane concentration in probable AD. Neurology.

[R33] Pei JJ, Hugon J (2008). mTOR-dependent signalling in Alzheimer’s disease. J Cell Mol Med.

[R34] Peng T, Xiang J, Tian Y, Tang X, Wang L, Gao L, Luo OJ, Huang L, Chen G (2025). Lycium barbarum glycopeptide ameliorates aging phenotypes and enhances cardiac metabolism by activating the PINK1/Parkin-mediated mitophagy pathway in D-galactose-induced mice. Exp Gerontol.

[R35] Pennisi M, Crupi R, Di Paola R, Ontario ML, Bella R, Calabrese EJ, Crea R, Cuzzocrea S, Calabrese V (2017). Inflammasomes, hormesis, and antioxidants in neuroinflammation: Role of NRLP3 in Alzheimer disease. J Neurosci Res.

[R36] Prakash P, Lantz TC, Jethava KP, Chopra G (2019). Rapid, refined, and robust method for expression, purification, and characterization of recombinant human amyloid beta 1-42. Methods Protoc.

[R37] Qiao X, Li W, Zheng Z, Liu C, Zhao L, He Y, Li H (2024). Inhibition of the HMGB1/RAGE axis protects against cisplatin-induced ototoxicity via suppression of inflammation and oxidative stress. Int J Biol Sci.

[R38] Sun Z, Liu J, Chen Z, So KF, Hu Y, Chiu K (2024). Lycium barbarum extract enhanced neuroplasticity and functional recovery in 5xFAD mice via modulating microglial status of the central nervous system. CNS Neurosci Ther.

[R39] Sutherland GT, Chami B, Youssef P, Witting PK (2013). Oxidative stress in Alzheimer’s disease: Primary villain or physiological by-product?. Redox Rep.

[R40] Takeda A, Tamano H (2021). Alzheimer’s disease pathogenesis focused on intracellular Zn2+ toxicity and its defense strategy. Nihon Yakurigaku Zasshi.

[R41] Tian GY, Chen W, Feng YC (1995). Isolation, purification and properties of LbGP and characterization of its glycan-peptide bond. Acta Biochim Biophys Sin.

[R42] Tremblay RG, Sikorska M, Sandhu JK, Lanthier P, Ribecco-Lutkiewicz M, Bani-Yaghoub M (2010). Differentiation of mouse Neuro 2A cells into dopamine neurons. J Neurosci Methods.

[R43] Urrutia PJ, Hirsch EC, González-Billault C, Núñez MT (2017). Hepcidin attenuates amyloid beta-induced inflammatory and pro-oxidant responses in astrocytes and microglia. J Neurochem.

[R44] Wang C, Chen S, Guo H, Jiang H, Liu H, Fu H, Wang D (2022). Forsythoside A mitigates Alzheimer’s-like pathology by Inhibiting ferroptosis-mediated neuroinflammation via Nrf2/GPX4 axis activation. Int J Biol Sci.

[R45] Wang X, Jia J (2023). Magnolol improves Alzheimer’s disease-like pathologies and cognitive decline by promoting autophagy through activation of the AMPK/mTOR/ULK1 pathway. Biomed Pharmacother.

[R46] Xiong M, Peng J, Zhou S, Gao Q, Lu J, Ou C, Song H, Peng Q (2025). Lycium barbarum L.: a potential botanical drug for preventing and treating retinal cell apoptosis. Front Pharmacol.

[R47] Xu S, Zhang Z, Zhou X, Liao Y, Peng Z, Meng Z, Nüssler AK, Ma L, Xia H, Liu L, Yang W (2025). Gouqi-derived Nanovesicles (GqDNVs) promoted MC3T3-E1 cells proliferation and improve fracture healing. Phytomedicine.

[R48] Yang SJ, Kim J, Lee SE, Ahn JY, Choi SY, Cho SW (2017). Anti-inflammatory and anti-oxidative effects of 3-(naphthalen-2-yl(propoxy)methyl)azetidine hydrochloride on β-amyloid-induced microglial activation. BMB Rep.

[R49] Zhang L, Wang H, Xu J, Zhu J, Ding K (2014). Inhibition of cathepsin S induces autophagy and apoptosis in human glioblastoma cell lines through ROS-mediated PI3K/AKT/mTOR/p70S6K and JNK signaling pathways. Toxicol Lett.

[R50] Zhao H, Alexeev A, Chang E, Greenburg G, Bojanowski K (2005). Lycium barbarum glycoconjugates: effect on human skin and cultured dermal fibroblasts. Phytomedicine.

[R51] Zheng C, Zhu R, Sun Z, Liu J, So KF, Chiu K (2022). Effects of Goji with different origins or different extraction methods on primary mixed glial cells. Chin Sci Bull.

[R52] Zheng H, Sun H, Cai Q, Tai HC (2024). The enigma of tau protein aggregation: mechanistic insights and future challenges. Int J Mol Sci.

[R53] Zheng J, Luo Z, Chiu K, Li Y, Yang J, Zhou Q, So KF, Wan QL (2023). Lycium barbarum glycopetide prolong lifespan and alleviate Parkinson’s disease in Caenorhabditis elegans. Front Aging Neurosci.

